# Paraneoplastic Opsoclonus Without Myoclonus Secondary to High-Grade Serous Carcinoma in an Adult

**DOI:** 10.7759/cureus.111379

**Published:** 2026-06-23

**Authors:** Caleb Bowman, Ibrahim Mustafa

**Affiliations:** 1 Medicine, Carle Illinois College of Medicine, Urbana, USA; 2 Neurology, Carle Illinois College of Medicine, Urbana, USA

**Keywords:** immunotherapy, opsoclonus, opsoclonus-myoclonus syndrome, paraneoplastic syndrome, serous carcinoma

## Abstract

Opsoclonus is a rare disorder characterized by chaotic, multidirectional eye movements and most commonly presents as part of the opsoclonus-myoclonus syndrome (OMS). Although typically described in pediatric populations, particularly in association with neuroblastoma, adult presentations are exceptionally rare and are most often paraneoplastic or infectious in origin. We describe the case of a woman in her 60s who developed isolated opsoclonus associated with a newly diagnosed poorly differentiated metastatic high-grade serous carcinoma. The clinical course, diagnostic evaluation, antibody profiling, and immunomodulatory treatments, including intravenous immunoglobulin (IVIG), corticosteroids, and plasmapheresis, are reviewed. This case highlights the diagnostic challenges and therapeutic considerations associated with adult-onset paraneoplastic opsoclonus.

## Introduction

Opsoclonus is an ocular motility disorder characterized by rapid, spontaneous, uncontrolled back-and-forth eye movements (saccades) that oscillate about the midline without fixation and without intersaccadic intervals. Chaotic movements occurring in multiple planes (vertical, horizontal, and torsional) are a defining feature of opsoclonus, distinguishing it from ocular flutter, which occurs exclusively in the horizontal plane [1]. Although some cases occur in isolation, opsoclonus is most commonly described in the literature as part of a syndrome associated with myoclonus. Opsoclonus-myoclonus syndrome (OMS) is a rare disorder with an estimated incidence of 0.18 cases per million population [2]. The syndrome is more common in children, whereas adult cases are even rarer and are generally reported only in case reports or small case series.

Most cases of OMS are believed to be immune-mediated. Many are attributed to paraneoplastic syndromes, most notably neuroblastoma in children. In adults, OMS has been associated with small cell lung cancer (SCLC), breast carcinoma, ovarian teratoma, and less frequently with lymphoma, malignant melanoma, and other neoplasms of the respiratory, gynecologic, urologic, gastrointestinal, and hematologic systems [3-6]. To the authors' knowledge, based on an extensive review of the literature, only two cases associated with serous carcinoma have been reported, both originating from the ovary [7,8]. Opsoclonus has also been described as a post-infectious syndrome, while other reported causes include systemic autoimmune diseases, toxic exposures, and medications. A remaining subset of cases is considered idiopathic [4]. Known associated antibodies include anti-Hu (ANNA-1), anti-Ri (ANNA-2), anti-N-methyl-D-aspartate (NMDA) receptor antibodies, anti-voltage-gated calcium channel antibodies, anti-Kelch-like protein 11 antibodies, anti-human natural killer 1 (HNK-1) antibodies, and anti-glycine receptor antibodies [3,9,10].

Historically, treatment strategies have focused on immunosuppression and the removal of pathogenic antibodies. High-dose corticosteroids are generally considered first-line therapy, and intravenous immunoglobulin (IVIG) is frequently used in conjunction with corticosteroids. Rituximab has demonstrated efficacy in refractory cases, whereas plasmapheresis may be beneficial when rapid symptom control is required. Cyclophosphamide and mycophenolate mofetil have been used for long-term immunosuppressive therapy. Benzodiazepines, particularly clonazepam, may also provide symptomatic relief [4].

The present patient's opsoclonus occurred secondary to an exceptionally rare etiology, metastatic high-grade serous carcinoma. Furthermore, testing was negative for the paraneoplastic antibodies most commonly associated with OMS, including ANNA-1, ANNA-2, and NMDA receptor antibodies. Nevertheless, she demonstrated a favorable clinical response to conventional immunomodulatory therapies, including IVIG and high-dose corticosteroids.

## Case presentation

History

A woman in her 60s presented to the emergency department with acute-onset vertigo and left-sided ear pain that began when clearing her Eustachian tube during airplane travel. She was initially evaluated with a computed tomography (CT) scan of the head and started empirically on oral antibiotics for presumed otitis media. After her discharge, a urine culture revealed a urinary tract infection caused by *Escherichia coli*, prompting a change in antimicrobial therapy to provide cross-coverage.

Over the subsequent days, she developed worsening symptoms, including persistent and worsening vertigo, intractable emesis, and a rapid decline in mobility due to these symptoms. On return to care, she was unable to ambulate independently. Notably, she denied other neurological symptoms of muscle twitching, weakness, headache, numbness, seizures, vision, or hearing changes. Her past medical history included essential hypertension, hypercholesterolemia, and panic disorder without agoraphobia. She had no smoking history and consumed fewer than three standard alcoholic beverages in a typical week.

Physical examination

She was visibly distressed and anxious, with intermittent severe systolic hypertension (as high as 200 mmHg). The remainder of her vital signs were within normal limits. Ophthalmologic examination revealed spontaneous, multidirectional, high-frequency, nonrhythmic saccadic eye movements consistent with opsoclonus (Video 1). Visual acuity and fundal examination were not feasible due to frequent eye movements. Muscle strength, sensation, coordination, and mental status were preserved, though she exhibited mild generalized hyporeflexia. No myoclonus or ataxia was present.

**Video 1:** Video demonstrating the patient's ocular movements consistent with opsoclonus during examination of extraocular movements using an H-pattern finger-tracking test Note: Written informed consent for the publication of the video, including identifiable facial images, in this open-access article was obtained from the patient.

Diagnostics

Basic laboratory analysis revealed a mild leukocytosis with neutrophil predominance and a hypokalemic metabolic alkalosis consistent with emesis. Results of extensive autoimmune (Table 1) and infectious (Table 2) laboratory studies are shown. Lumbar puncture was deferred due to high suspicion of a paraneoplastic process and absence of meningeal signs.

**Table 1:** Serum laboratory test results for autoimmune antibodies ANA: antinuclear antibody, GAD65: glutamic acid decarboxylase 65, c-ANCA (PR3): cytoplasmic antineutrophil cytoplasmic antibody (proteinase 3), p-ANCA (MPO): perinuclear anti-neutrophil cytoplasmic antibody (myeloperoxidase), VGKC: voltage-gated potassium channel, AChR: acetylcholine receptor, VGCC: voltage-gated calcium channel, ANNA: antineuronal nuclear antibody, AGNA: anti-glial nuclear antibody, CRMP5: collapsin response mediator protein-5, PCA-Tr: Purkinje cell cytoplasmic antibody type Tr, LGI1: leucine-rich glioma-inactivated 1, CASPR2: contactin-associated protein-like 2, DPPX: dipeptidyl-peptidase-like protein-6, NMDA: N-methyl-D-aspartate receptor, GABA-B-R: gamma-aminobutyric acid type B receptor, mGluR1: metabotropic glutamate receptor 1, IgLON5: immunoglobulin-like cell adhesion molecule 5, NIF: negative inspiratory force, GFAP: glial fibrillary acidic protein, TRIM46: tripartite motif containing 46, PDE10A: phosphodiesterase 10A.

Antibody test	Result
Anti-SSA	Positive (4.1, Ref <1.0)
Anti-SSB	Negative
ANA	Positive (1:1280, Ref 1:40)
GAD65	Positive (0.15 nmol/L, Ref <0.02)
c-ANCA (PR3)	Negative
p-ANCA (MPO)	Negative
VGKC	Positive (155 pmol/L, Ref <80)
AChR ganglionic	Negative
VGCC P/Q	Negative
VGCC N	Negative
Striated muscle	Negative
Amphiphysin	Negative
ANNA-1	Negative
ANNA-2	Negative
ANNA-3	Negative
AGNA/SOX1	Negative
CRMP5	Negative
CV2	Negative
PCA-1 (Yo)	Negative
PCA-2	Negative
PCA-Tr (DNER)	Negative
LGI1	Negative
CASPR2	Negative
DPPX	Negative
AMPA-R	Negative
NMDA-R	Negative
GABA-B-R	Negative
mGluR1	Negative
IGLON5	Negative
Neurochondrin	Negative
NIF	Negative
GFAP	Negative
SEPTIN-7	Negative
TRIM46	Negative
PDE10A	Negative

**Table 2:** Serum laboratory test results for infectious antibodies (Ab) and antigens (Ag)

Test	Result
Treponema pallidum AB	Negative
Lyme AB	Negative
Hepatitis B core AB	Positive
Hepatitis B surface AB	Positive (>1000, Ref <5)
Hepatitis B surface Ag	Negative
Hepatitis C AB	Negative
HIV AB	Negative
HIV Ag	Negative

**Figure 1:**
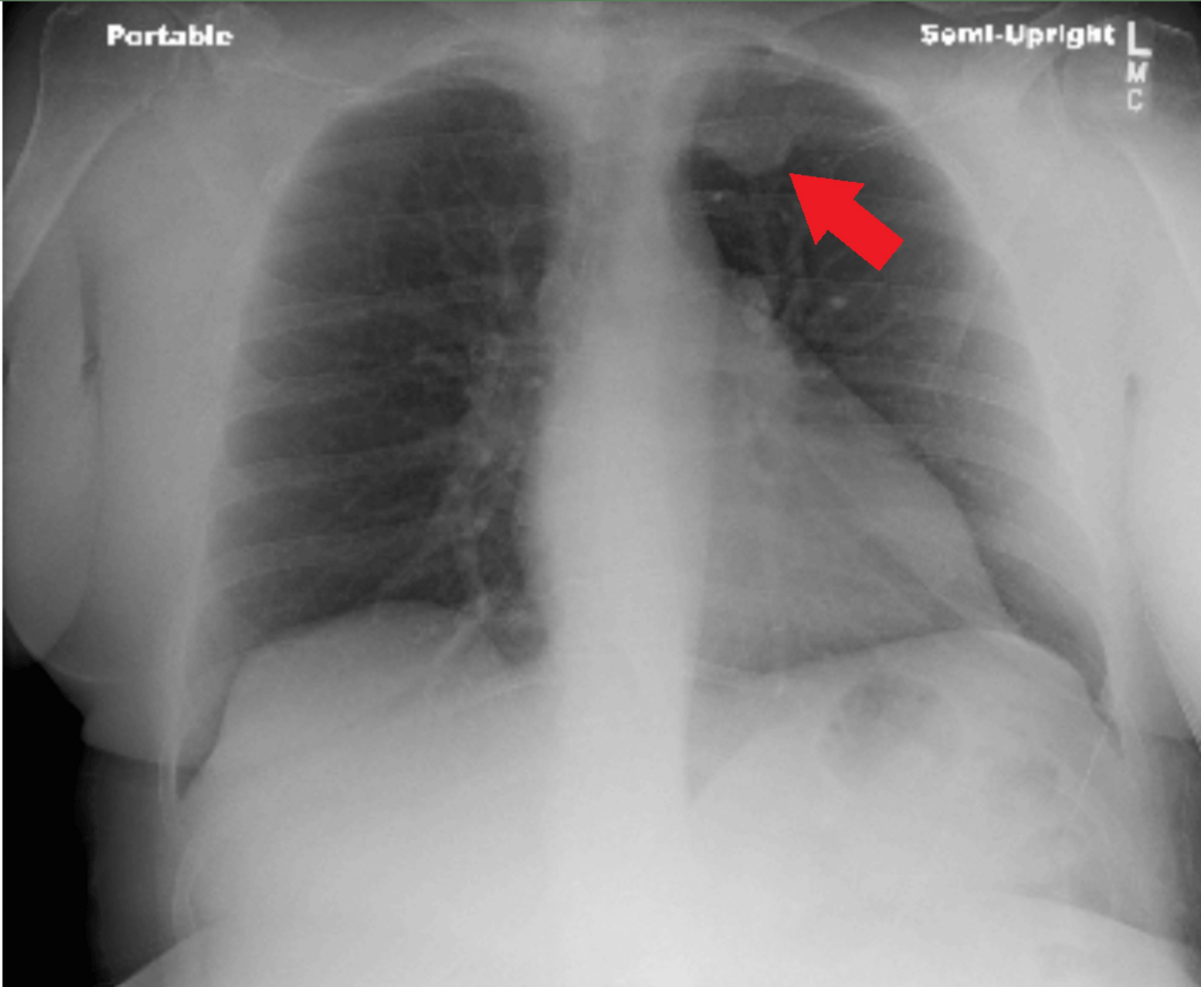
Chest radiograph demonstrating a left apical lung mass

**Figure 2:**
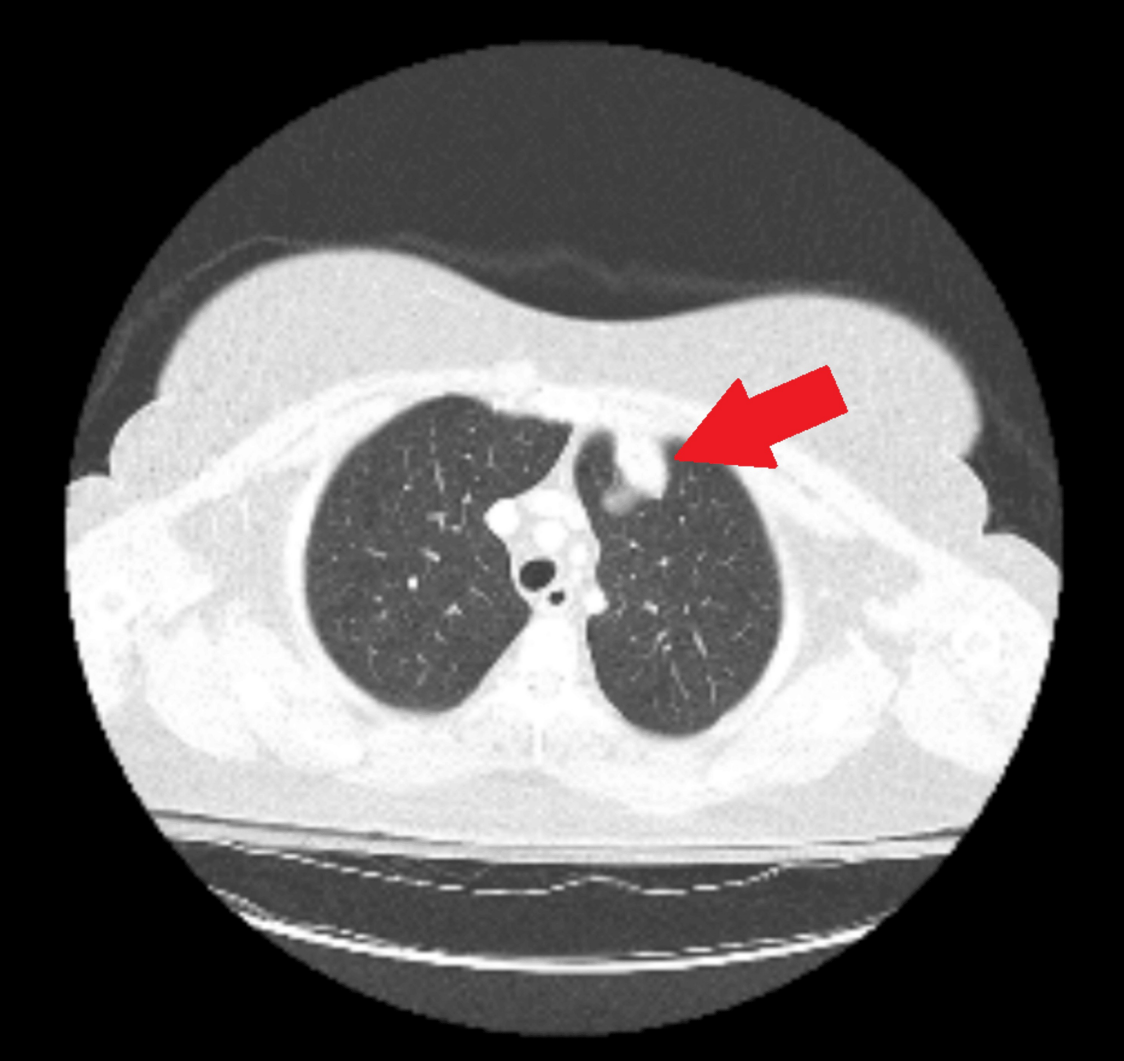
Computed tomography (CT) of the chest showing the patient’s left apical lung mass

**Figure 3:**
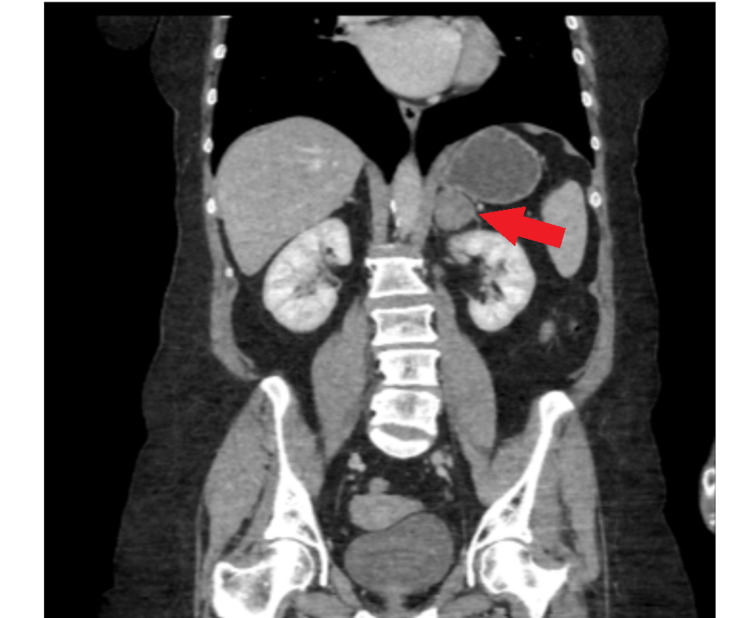
Computed tomography (CT) of the abdomen showing the left adrenal mass

**Figure 4:**
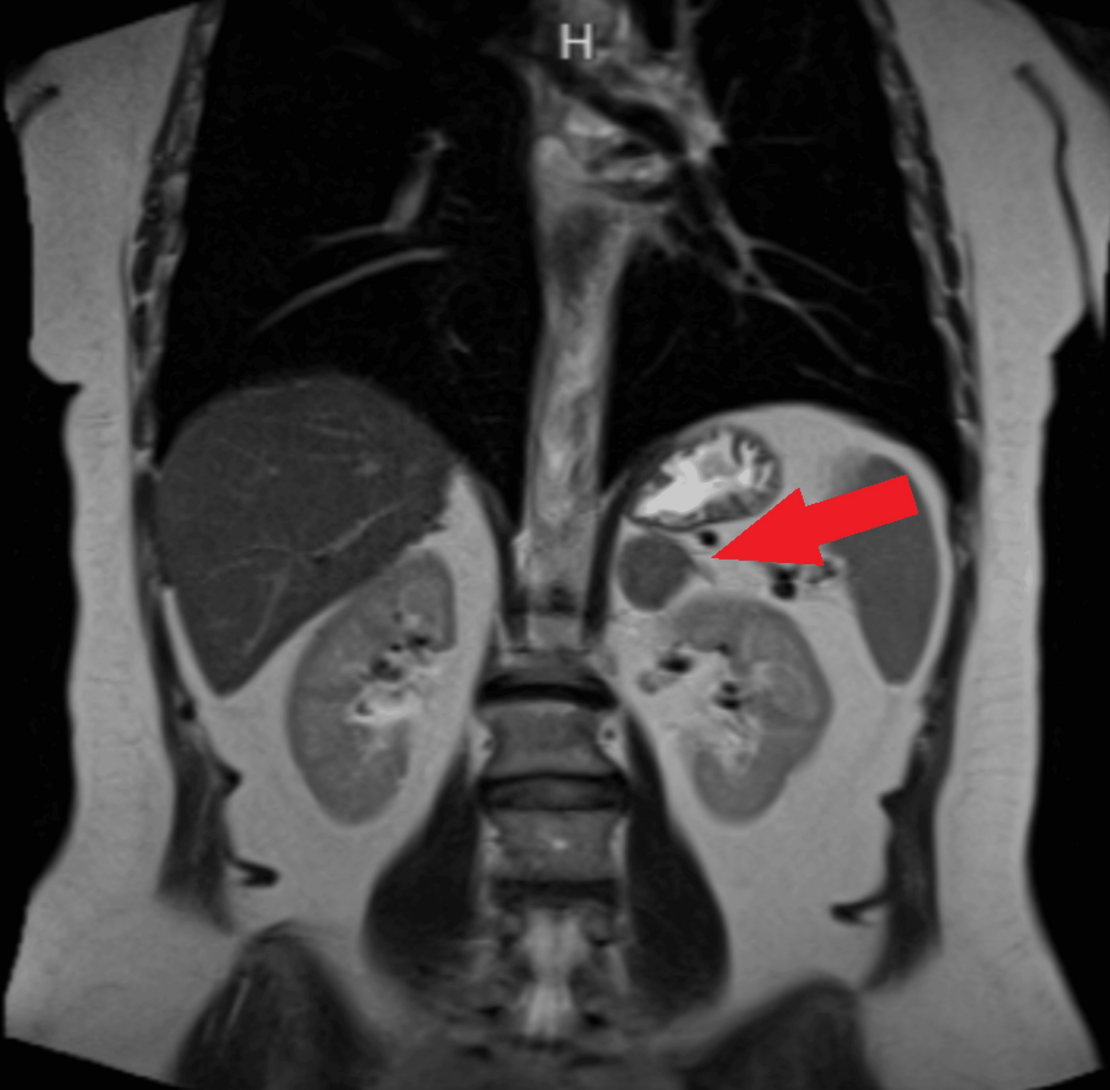
Magnetic resonance imaging (MRI) of the abdomen showing the patient’s left adrenal mass

An initial chest radiograph (Figure 1) revealed a 4-5 cm mass in the left lung apex. Follow-up CT (Figure 2 and Figure 3) and magnetic resonance imaging (MRI) (Figure 4) of the chest, abdomen, and pelvis identified additional lesions: a left adrenal mass, a sclerotic focus in the right sacrum, a probable hepatic hemangioma, and multiple enlarged retroperitoneal and mediastinal lymph nodes. Initial MRI of the brain was unremarkable. Immunohistochemistry of tumor cells from lung lesion biopsy was positive for cytokeratin 7 (CK7), pankeratin, and PAX8, and negative for CK20, TTF-1, p40, and other markers, most consistent with a poorly differentiated metastatic serous carcinoma. A later positron emission tomography (PET) scan was equivocal for hypermetabolic activity in the pelvis, and MRI revealed no lesions. The pelvic exam was unremarkable, and CA-125 was within normal limits.

Clinical course and treatment

The patient was admitted and began symptomatic therapy with oral ondansetron (4 mg) and meclizine (25 mg) for nausea. Intravenous lorazepam (1 mg three times daily) was initiated to help alleviate distress from opsoclonus. These measures effectively palliated nausea and distress without remission. A three-day course of intensive IVIG (1000 mg/kg/day) was administered, resulting in mild improvement of ocular symptoms and partial recovery of ambulation with assistance.

Within days, her clinical status deteriorated with the onset of dysphagia, fluctuating mentation, and worsening autonomic instability. MRI of the brain remained unremarkable. She was started on a regimen of systemic chemotherapy including carboplatin, pemetrexed (Alimta), and pembrolizumab (Keytruda), with the intent of mitigating tumor burden and related paraneoplastic immune activation. A second course of IVIG (400 mg/kg/day for five days) was followed by high-dose intravenous methylprednisolone (1 g/day for five days). Benzodiazepine therapy was transitioned to clonazepam for sustained symptomatic relief. Electroencephalography demonstrated moderate diffuse encephalopathy.

The patient was subsequently transferred to a quaternary care center where she underwent five sessions of plasmapheresis. This intervention led to marked improvements in her cognition with return to baseline, although resolution of opsoclonus was incomplete. She was discharged to a skilled nursing facility for ongoing supportive care and rehabilitation.

## Discussion

Opsoclonus is a rare ocular motor disturbance most commonly observed in children as part of the OMS in a paraneoplastic or post-infectious setting. It is less commonly encountered in adults and rarely occurs in the absence of myoclonus. This case is particularly notable because of its association with a poorly differentiated metastatic high-grade serous carcinoma of unknown primary origin, an etiology for which only a few cases have been reported in the literature [7,8].

Although voltage-gated potassium channel (VGKC) and glutamic acid decarboxylase 65 (GAD65) antibodies are known to have relatively high false-positive rates [11], our patient's antibody profile, including positive ANA, SS-A, VGKC, and GAD65 antibodies in the absence of the classical paraneoplastic antibodies ANNA-1, ANNA-2, and NMDA receptor antibodies [3,9,10], suggests a broader autoimmune response that may have contributed to her clinical presentation. Laboratory testing also demonstrated evidence of prior hepatitis B infection, although the timing of infection could not be determined. While post-infectious OMS associated with hepatitis B has been described in the literature [4], a paraneoplastic etiology was considered more likely in light of the concurrent diagnosis of metastatic carcinoma. The subsequent development of additional neurological symptoms further supports the possibility of an underlying autoimmune encephalopathic process. Treatment focused on immunosuppression and antibody removal through corticosteroids, IVIG, and plasmapheresis, consistent with current approaches to immune-mediated opsoclonus [4].

Although the initial response to IVIG and corticosteroids was modest, plasmapheresis was associated with meaningful cognitive improvement. The limited improvement in ocular symptoms despite aggressive immunotherapy underscores the therapeutic challenges associated with adult-onset paraneoplastic opsoclonus.

## Conclusions

This case highlights the importance of prompt recognition of opsoclonus as a potential paraneoplastic syndrome, thereby prompting an evaluation for an underlying malignancy. In this patient, metastatic high-grade serous carcinoma of unknown primary origin was identified as the underlying cause, a previously described but exceedingly rare association. The progressive development of concomitant encephalopathic symptoms, together with the presence of multiple autoantibodies, is consistent with the existing literature describing opsoclonus as an autoimmune paraneoplastic phenomenon and suggests broad immunological activation. However, antibodies classically associated with this condition were absent. Further research into immunologic markers may improve diagnostic accuracy and prognosis in this rare and challenging condition. Standard immunosuppressive therapies, including corticosteroids, IVIG, and plasmapheresis, in conjunction with the initiation of chemotherapy, resulted in partial symptomatic remission in this case, underscoring the value of a multidisciplinary approach.

## References

[1] Grossman SN, Rucker JC (2023). Opsoclonus and ocular flutter: evaluation and management. Curr Opin Ophthalmol.

[2] Pang KK, de Sousa C, Lang B, Pike MG (2010). A prospective study of the presentation and management of dancing eye syndrome/opsoclonus-myoclonus syndrome in the United Kingdom. Eur J Paediatr Neurol.

[3] Du H, Cai W (2022). Opsoclonus-myoclonus syndrome associated with neuroblastoma: Insights into antitumor immunity. Pediatr Blood Cancer.

[4] Klaas JP, Ahlskog JE, Pittock SJ (2012). Adult-onset opsoclonus-myoclonus syndrome. Arch Neurol.

[5] Jones AA, Chen T (2022). Delayed opsoclonus-myoclonus syndrome after ovarian teratoma resection. J Neuroophthalmol.

[6] Grubbs J Jr, Trobe JD, Fisher-Hubbard A (2016). Opsoclonus-myoclonus syndrome in primary central nervous system lymphoma. J Neuroophthalmol.

[7] Stewart KT, Lee JS, Stuart G (2019). Paraneoplastic opsoclonus-myoclonus syndrome as a presentation of high grade serous ovarian cancer. Gynecol Oncol Rep.

[8] Rubio Nazábal E, Marey López J, Avarez Pérez P, López Facal S, Alonso Magdalena L (2003). Opsoclonus-myoclonus syndrome in patient with ovarian cancer. Ann Med Interna (Madrid).

[9] Armangué T, Sabater L, Torres-Vega E (2016). Clinical and immunological features of opsoclonus-myoclonus syndrome in the era of neuronal cell surface antibodies. JAMA Neurol.

[10] Fonseca E, Varas R, Godoy-Santín J, Valenzuela R, Sandoval P (2021). Opsoclonus-myoclonus syndrome associated with anti Kelch-like protein-11 antibodies in a young female patient without cancer. J Neuroimmunol.

[11] Grain R, Lally J, Stubbs B, Malik S, Nicholson TR, Murray RM, Gauhgran F (2017). Autoantibodies against voltage-gated potassium channel and glutamic acid decarboxylase in psychosis: A systematic review, meta-analysis, and case series. Psychiatry Clin Neurosci.

